# Quality of life of female and male vegetarian and vegan endurance runners compared to omnivores – results from the NURMI study (step 2)

**DOI:** 10.1186/s12970-018-0237-8

**Published:** 2018-07-17

**Authors:** Patrick Boldt, Beat Knechtle, Pantelis Nikolaidis, Christoph Lechleitner, Gerold Wirnitzer, Claus Leitzmann, Thomas Rosemann, Katharina Wirnitzer

**Affiliations:** 1 Faculty of Medicine, University of Gießen, Gießen, Germany; 2 Institute of Primary Care, University of Zurich, Zurich, Switzerland; 3 Exercise Physiology Laboratory, Nikaia, Greece; 4 ITEG, Innsbruck, Austria; 5 AdventureV & change2V, Stans, Austria; 6 Institute of Nutrition, University of Gießen, Gießen, Germany; 7 Institute of Primary Care, University of Zurich, Zurich, Switzerland; 8 Centre for Research and Knowledge Management, Pedagogical University Tyrol, Feldstraße 1/II, 6010 Innsbruck, Austria; 9 Department of Sport Science, University of Innsbruck, Innsbruck, Austria

**Keywords:** Vegetarian, Vegan, Diet, Nutrition, Marathon running, Quality of life, Life satisfaction, WHOQOL-BREF

## Abstract

**Background:**

Health-related effects of a vegetarian or vegan diet are known to support parameters positively affecting exercise performance in athletes, whereas knowledge about psyche and wellbeing is sparse. Therefore, the aim of the Nutrition and Running High Mileage (NURMI) Study (Step 2) was to compare Quality of Life (QOL) scores among endurance runners following a vegetarian or vegan diet against those who adhere to an omnivorous diet.

**Methods:**

The study was conducted following a cross-sectional design. A total of 281 recreational runners (159 women, 122 men) completed the WHOQOL-BREF questionnaire consisting of the domains physical health, psychological wellbeing, social relationships and environment, which generates scores on a scale from 4 to 20. Data analysis was performed using ANOVA.

**Results:**

It was found that 123 subjects followed an omnivorous diet and 158 adhered to a vegetarian/vegan diet. There were 173 runners who met the inclusion criteria (‘NURMI-Runners’), among them 103 half-marathoners and 70 marathoners and ultramarathoners, as well as 108 10 km runners as control group. Overall QOL scores were high (~ 16.62 ± 1.91). Men had higher scores than women due to high scores in the physical health and psychological well-being dimensions. Adhering to an omnivorous diet affected environment scores for women and social relationships scores for men. A minor effect concerning race distance was observed in women, where half-marathoners had a higher environmental score than 10-km runners. A moderate diet×race distance interaction on environment scores was shown for men.

**Conclusions:**

The results revealed that endurance runners had a high QOL regardless of the race distance or diet choice. These findings support the notion that adhering to a vegetarian or vegan diet can be an appropriate and equal alternative to an omnivorous diet.

**Trial registration:**

ISRCTN73074080. Registered 12th June 2015, retrospectively registered.

## Background

In the past 15 years, the number of participants in endurance running events, such as marathon or half-marathon races, has consistently been at a high level [1, 2]. More and more athletes among these adhere to a vegetarian or vegan diet [3, 4].

Health-related effects of a vegetarian or vegan diet have been found in athletes and are known to support parameters that positively affect exercise performance, such as physical fitness, resilience to chronic diseases, and weight control [5–7]. However, the knowledge about psychological and personal well-being is sparse. In order to generate an impression of an individual’s life situation, meaning her/his needs, problems, concerns and emotional state, it can be appropriate to measure Quality of Life (QOL): a multidimensional concept that measures life satisfaction, including family, physical health, education, employment, wealth, religious beliefs, finance and environment factors [8, 9].

Variables that affect QOL include sex, dietary habits and physical activity [10–14]. The investigation of the impact of sex on QOL has yielded various results. Whereas in some studies higher QOL scores have been found in men [15–17], it has also been reported that women have higher scores [14]. The dimension of social relationships especially has had higher scores in female subjects [18]. In terms of sex differences in QOL-scores in endurance runners, no data has been made available yet.

The impact of diet choice on QOL has been investigated in the general population. A high QOL in general has been reported for both vegetarians [13] and vegans [10], as well as the beneficial effects of a meatless diet rich in fruits and vegetables on the QOL dimensions of ‘depression’ [19], ‘anxiety’ [20] and ‘felt stress’ [21, 22]. The rationale for this interconnectedness is that being a vegetarian or vegan is both a dietary habit and a lifestyle [23]. For many, vegetarianism and veganism are philosophies of how life should be lived and hence they are connected with certain characteristics, such as being health-conscious, liberal and having a humanistic view of the world [24]. As vegetarian and vegan dietary patterns are frequently considered in the dietary strategies of athletes [6], the purpose of the present study was to investigate to what extent findings from the general population apply to endurance athletes.

Similar results have been found for physical activity. It has been shown that physical activity in general can lead to a high QOL [12, 25]. This has been confirmed by other studies investigating strength training [26], cycling [27] and musculoskeletal fitness [28]. As ‘physical health’ is an important requirement for life satisfaction, the synergistic effects of persistent adherence to a healthy diet and regular sport necessarily strongly influences QOL [23]. Further beneficial effects of an active lifestyle have been shown for numerous facets of QOL, such as ‘life satisfaction’ [29], ‘sleep architecture’ [30], ‘felt stress’ [31], ‘anxiety’ [32] and ‘depression’ [33].

All in all, some knowledge exists in terms of QOL and its interconnectedness with sex, diet choice and physical activity for the general population, suggesting there may be positive effects of a vegetarian and vegan diet on QOL. However, the data in terms of endurance runners and QOL is sparse. Therefore, in the Nutrition and Running High Mileage (NURMI) Study Step 2 we focused on the QOL of endurance runners, in particular in half-marathoners and marathoners. In the context of a rising number of athletes following a vegetarian or vegan diet [3, 4] and a lack of scientific literature concerning these groups, the aim of the study was to investigate QOL in endurance runners adhering to a vegetarian or vegan diet and compare them to endurance runners following a mixed diet.

Based on the findings from the general population, we hypothesized that QOL of omnivorous and vegetarian/vegan endurance runners would be similar. Hence, a vegetarian or vegan diet could be an equivalent alternative to an omnivorous diet for endurance athletes.

## Methods

### Experimental approach to the problem

We assessed QOL using the WHOQOL-BREF [World Health Organization Quality of Life Assessment- brief (French: *bref*) version]. The WHOQOL-BREF is a shorter version of the original instrument that may be more convenient for use in large research studies or clinical trials [34]. The WHOQOL-BREF’s validity is well established and has been confirmed by a number of studies [9, 35, 36].

The WHOQOL-BREF instrument comprises 26 items, which measure the following broad domains: physical health (i.e. activities of daily living, dependence on medicinal substances and medical aids, energy and fatigue, mobility, pain and discomfort, sleep and rest, work capacity; DOM 1), psychological well-being (i.e. bodily image and appearance, negative feelings, positive feelings, self-esteem, spirituality/religion/personal beliefs, thinking, learning, memory and concentration; DOM 2), social relationships (i.e. personal relationships, social support, sexual activity; DOM 3) and environment (i.e. financial resources, freedom, physical safety and security, health and social care: accessibility and quality, home environment, opportunities for acquiring new information and skills, participation in and opportunities for recreation/leisure activities, physical environment (i. e. pollution/noise/traffic/climate, transport; DOM 4).

Each item was rated on a 5-point Likert scale. The typical Likert scale is a 5-point ordinal scale used by respondents to rate the degree to which they agree or disagree with a statement (i.e. higher scores denote stronger agreement or disagreement, respectively).

Afterwards, four domain scores were derived. Raw domain scores for the WHOQOL were transformed to a 4–20 score and scaled in a positive direction (i.e. higher scores denote higher QOL). The mean score of items within each domain was used to calculate the domain score [34].

### Subjects

The NURMI Study was conducted in three steps following a cross-sectional design. We recruited endurance runners mainly from German-speaking countries, such as Germany, Austria and Switzerland. In addition, we approached people from all over Europe. The subjects were contacted mainly via social media, websites of the organizers of marathon events, online running communities, email-lists, runners’ magazines as well as magazines for health, vegetarian and/or vegan nutrition and lifestyle, sports fairs, fairs on vegetarian and vegan nutrition and lifestyle, and through personal contacts.

The study protocol [4] was approved by the ethics board of St. Gallen, Switzerland on May 6, 2015 (EKSG 14/145). The trial registration number is ISRCTN73074080.

### Procedures

The participants completed an online survey within the NURMI Study Step 2, provided in German and English, which was available on https://www.nurmi-study.com/en from February 1st 2015 until December 31st 2015.

The survey started with a written description of the procedure and participants gave their informed consent to take part in the study. Afterwards, they completed the WHOQOL-BREF questionnaire (for further information see below) including questions concerning physical health, psychological well-being, social relationships and environment. In addition, we asked for age, sex and preferred diet.

For successful participation, the following criteria were required: written informed consent (1), at least 18 years of age (2), WHOQOL-BREF questionnaire completed (3), successful participation in a running event of either half-marathon or marathon distance in the past two years (4). Incomplete and inconsistent data sets were eliminated. Those who met all inclusion criteria but named a 10-km race as their running event were kept as controls. In the following they are called ‘10-km control group’, whereas those who met the inclusion criteria to the full extent are referred to as ‘NURMI-Runners’.

Participants were classified into two diet groups: omnivorous diet (commonly known as Western diet, no dietary restrictions) versus vegetarian (no meat)/vegan (no products from animal sources, such as meat, fish, milk and dairy products, eggs and honey) diet [5]. Moreover, they were categorized into three race distances: 10 km, half-marathon and marathon/ultramarathon.

### Statistical analyses

The statistical software IBM SPSS version 23.0 (SPSS, Chicago, USA) and GraphPad Prism version 7.0 (GraphPad Software, San Diego, USA) performed all statistical analyses. The Kolmogorov-Smirnoff test of normality and visual inspection of normal Q-Q plots examined the normality of all variables. Mean values and standard deviation (SD) were calculated for all variables. The student t-test examined sex differences in the four domains of WHOQOL and Cohen’s d (*d* ≤ 0.2, trivial; 0.2 < *d* ≤ 0.6, small; 0.6 < *d* ≤ 1.2, moderate; 1.2 < *d* ≤ 2.0, large; and *d* > 2.0, very large) evaluated the magnitude of these differences. A two-way ANOVA, followed by a Bonferroni post-hoc analysis, examined the main effects of nutrition and race distance, the nutrition*race distance interaction on WHOQOL. The magnitude of differences in the ANOVA was evaluated using eta squared (η^2^) as trivial (η^2^ < 0.01), small (0.01 ≤ η^2^ < 0.06), moderate (0.06 ≤ η^2^ < 0.14) and large (η^2^ ≥ 0.14). The level of statistical significance was set at *p* ≤ 0.05.

## Results

A total of 317 endurance runners completed the survey, of whom 281 (159 women and 122 men) with a mean age of 40 ± 11 years remained after data clearance. Their countries of origin were Germany (*n* = 200), Switzerland (*n* = 14), Austria (*n* = 50) and some others (*n* = 17; Belgium, Brazil, Canada, Italy, Luxemburg, Netherlands, Poland, Spain, United Kingdom)*.*

With regard to dietary subgroups, 123 subjects followed an omnivorous diet and 158 adhered to a vegetarian/vegan diet. Concerning race distances, there were 173 NURMI-Runners (103 half-marathoners, 70 marathoners/ultramarathoners) and 108 members of the 10-km control group. Characteristics of our subjects are presented in Table 1.

**Table 1:** Anthropometric and Demographic Characteristics of the Subjects Displayed by Diet Group

		Total	Omnivorous	Vegetarian/Vegan	*p*
Number of Subjects		281 (100%)	123 (43.77%)	158 (56.23%)	
Sex	Female	159 (56.58%)	58 (47.15%)	101 (63.92%)	0.005
	Male	122 (43.42%)	65 (52.85%)	57 (36.08%)	
Mean Age (years)		40.00 ± 11.00	41.96 ± 11.02	38.26 ± 10.84	0.005
Race Distance					
Control Group	10 km	108 (38.43%)	43 (34.96%)	65 (41.14%)	0.561
NURMI-Runners	Half-Marathon	103 (36.65%)	47 (38.21%)	56 (35.44%)	
	Marathon/Ultramarathon	70 (24.91%)	33 (26.83%)	37 (23.42%)	
Mean Body Weight (kg)		65.62 ± 10.53	67.91 ± 10.78	63.85 ± 10.01	0.001
Mean Height (m)		1.72 ± 0.87	1.73 ± 0.08	1.72 ± 0.90	0.134
Mean BMI_CALC_ (kg/m^2^)		22.03 ± 2.49	22.55 ± 2.44	21.63 ± 2.45	0.002
Academic Qualification	No Qualification	1 (< 1%)	0 (< 1%)	1 (< 1%)	0.464
	Upper Secondary Education/Technical Qualification/GCSE or Equivalent	94 (33.45%)	45 (36.59%)	49 (31.01%)	
	A Levels or Equivalent	62 (22.06%)	32 (26.02%)	30 (18.99%)	
	University Degree/Higher Degree (*i. e.* doctorate)	96 (34.16%)	36 (29.27%)	60 (37.97%)	
	No Answer	28 (9.96%)	10 (8.13%)	18 (11.39%)	
Marital Status	Divorced/Separated	16 (5.69%)	3 (2.44%)	13 (8.23%)	0.004
	Married/Living with Partner	190 (67.62%)	94 (76.42%)	96 (60.76%)	
	Single	75 (26.69%)	26 (21.14%)	49 (31.01%)	
Country of Residence	Austria	50 (17.79%)	28 (22.76%)	22 (13.92%)	0.010
	Germany	200 (71.17%)	85 (69.11%)	115 (72.78%)	
	Switzerland	14 (4.98%)	8 (6.5%)	6 (3.8%)	
	Other	17 (6.05%)	2 (1.63%)	15 (9.49%)	

*Note*. Results are presented as mean ± SD*.* 10 km – 10 Kilometer Control Group. BMI_CALC_ – Body Mass Index (calculated). p – p-value for difference among groups

### Sex differences in quality of life

**Fig. 1:**
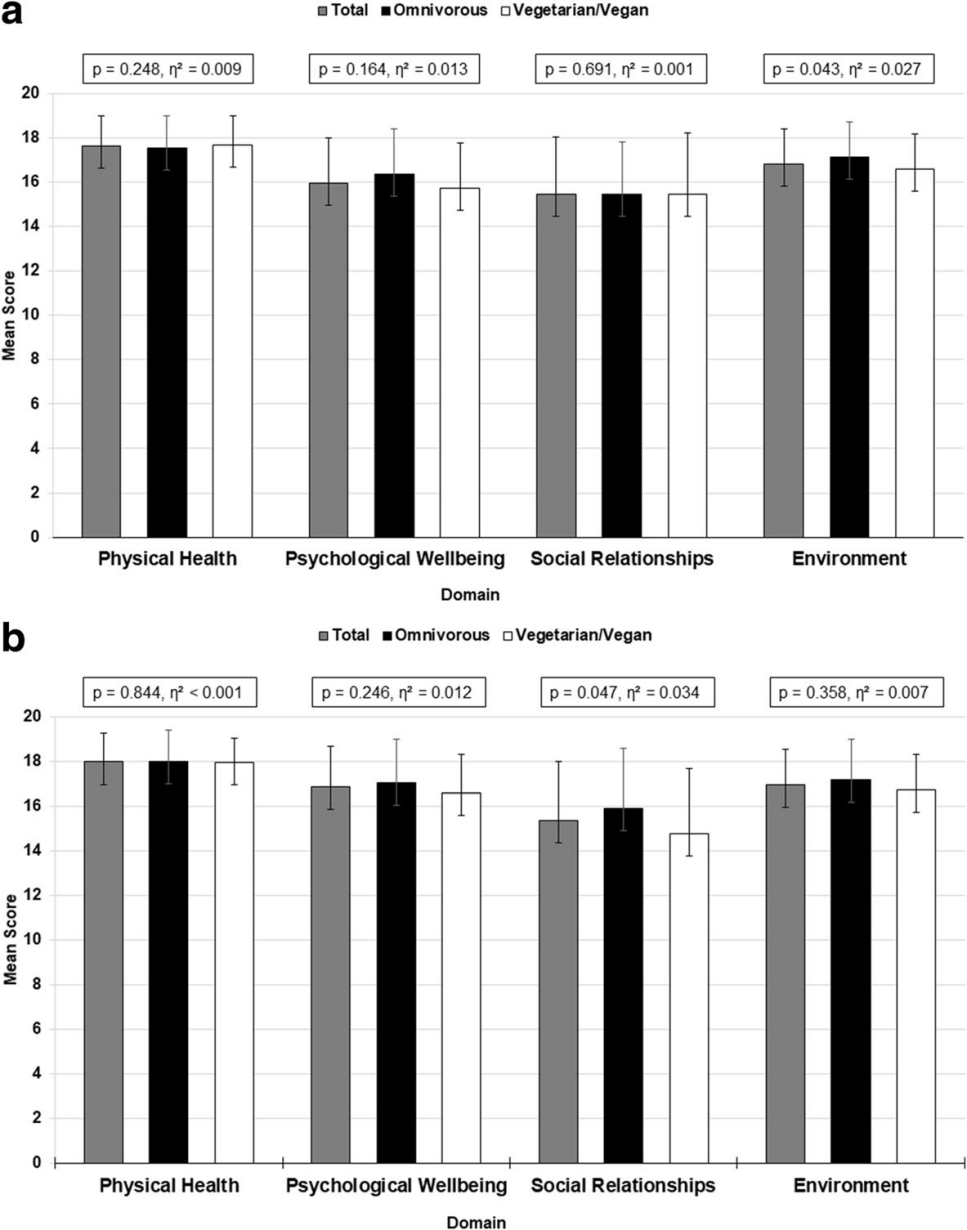
**a** Mean WHOQOL-BREF-Domain Scores of Women Displayed by Diet Group. *Note.* Results are presented as mean ± SD*.* p – *p*-value for differences between groups. η^2^ – effect size. **b**. Mean WHOQOL-BREF-Domain Scores of Men Displayed by Diet Group. *Note.* Results are presented as mean ± SD*.* p – *p*-value for differences between groups. η^2^ – effect size

**Fig. 2:**
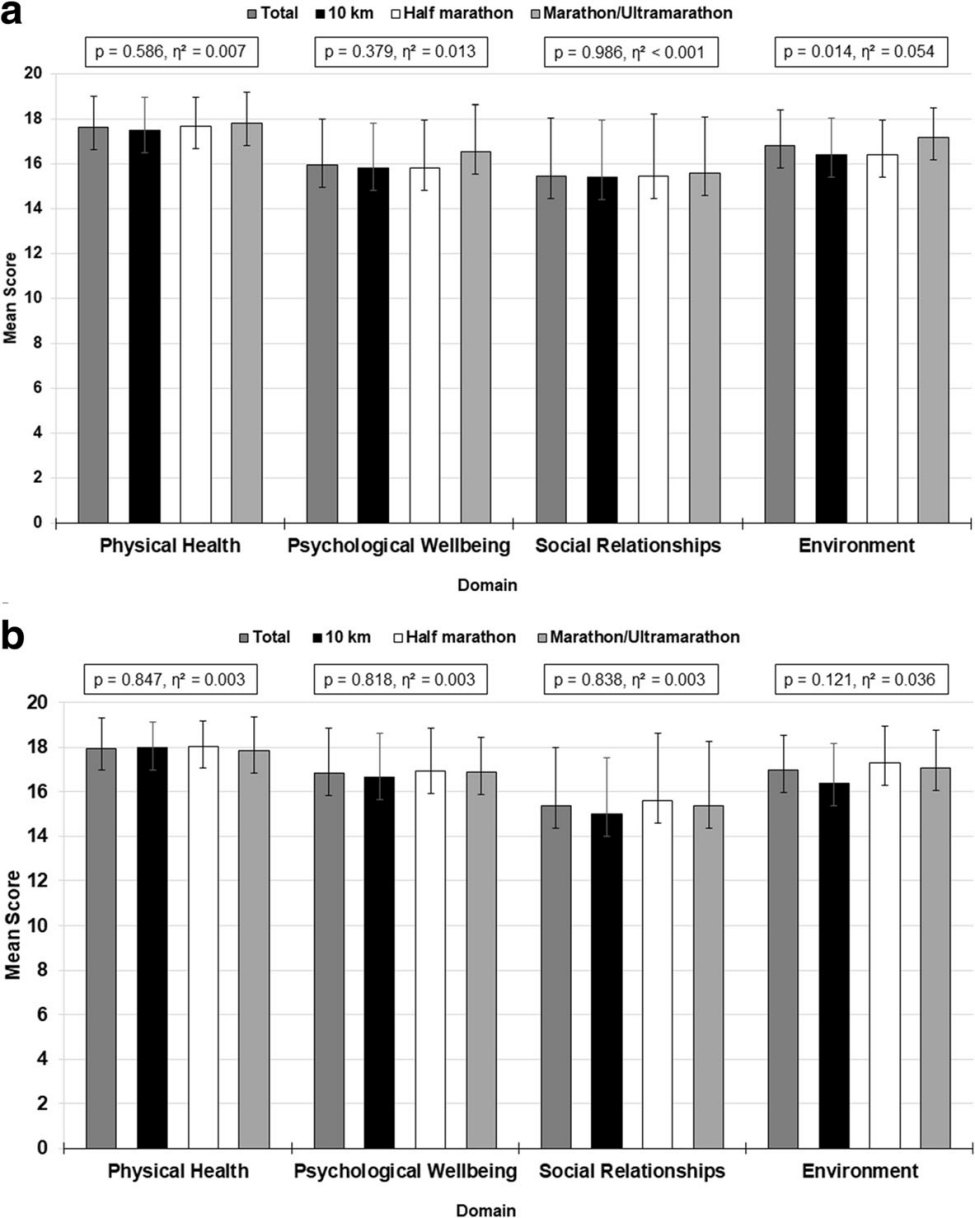
**a** Mean WHOQOL-BREF-Domain Scores of Women Displayed by Race Distance. *Note.* Results are presented as mean ± SD*.* p – *p*-value for differences between groups. η^2^ – effect size. **b** Mean WHOQOL-BREF-Domain Scores of Men Displayed by Race Distance. *Note.* Results are presented as mean ± SD*.* p – *p*-value for differences between groups. η^2^ – effect size

Scores for physical health were 17.6 ± 1.4 (85.13%) in women and 18.0 ± 1.3 (87.24%) in men, for psychological wellbeing 16.0 ± 2.1 (74.71%) and 16.8 ± 1.8 (80.16%), for social relationships 15.5 ± 2.6 (71.59%) and 15.4 ± 2.9 (70.97%), and for environment 16.8 ± 1.6 (80.05%) and 17.0 ± 1.7 (80.99%). Men had higher scores in physical health (*p* = 0.037, d = 0.26) and psychological wellbeing (*p* < 0.001, d = 0.45), but there were no differences with regard to social relationships counts (*p* = 0.761, d = 0.03) and environment scores (*p* = 0.445, d = 0.09) compared to women (Fig. 1a, b, 2a, b).

### Main effects of diet choice on quality of life

Scores for physical health were 17.5 ± 1.5 (84.6%) in female omnivorous runners, 18.0 ± 1.4 (87.4%) in male omnivorous runners, 17.7 ± 1.5 (85.4%) in female vegetarian/vegan runners and 17.9 ± 1.1 (87.0%) in male vegetarian/vegan runners. With regard to psychological wellbeing, mean scores were 16.4 ± 2.0 (77.3%) in female omnivorous runners, 17.0 ± 1.9 (81.5%) in male omnivorous runners, 15.7 ± 2.0 (73.3%) in female vegetarian/vegan runners and 16.6 ± 1.7 (78.6%) in male vegetarian/vegan runners. Social relationships scores were 15.5 ± 2.3 (71.7%) in female omnivorous runners, 15.9 ± 2.7 (74.4%) in male omnivorous runners, 15.5 ± 2.8 (71.6%) in female vegetarian/vegan runners and 14.7 ± 2.9 (67.1%) in male vegetarian/vegan runners. In terms of environment, mean scores were 17.2 ± 1.6 (82.2%) in female omnivorous runners, 17.2 ± 1.8 (82.3%) in male omnivorous runners, 16.6 ± 1.6 (78.8%) in female vegetarian/vegan runners and 16.7 ± 1.6 (79.6%) in male vegetarian/vegan runners (Fig. 1a and b).

No major effect of diet on physical health (*p* = 0.248, η^2^ = 0.009 and *p* = 0.844, η^2^ < 0.001), and psychological wellbeing (*p* = 0.164, η^2^ = 0.013 and *p* = 0.246, η^2^ = 0.012) in both sexes, on social relationships in women (*p* = 0.691, η^2^ = 0.001) or on environment in men (*p* = 0.358, η^2^ = 0.007) was observed. However, a minor effect of diet on social relationships in men (*p* = 0.047, η^2^ = 0.034) and environment in women (*p* = 0.043, η^2^ = 0.027) was shown with a higher score in the omnivorous diet **(**Fig. 1a**,** Fig. 1b**)**. Mean scores for each item are shown in Table 2.

**Table 2:** Mean Likert-Scores of the WHOQOLBREF-Items Displayed by Diet Group

Question	Total	Omnivorous	Vegetarian/Vegan	p	η^2^
How would you rate your Quality of Life? ^1^					
Women	4.33 ± 0.58	4.38 ± 0.59	4.30 ± 0.58	0.450	0.002
Men	4.48 ± 0.65	4.46 ± 0.74	4.49 ± 0.53		
How satisfied are you with your health? ^2^					
Women	4.09 ± 0.93	4.03 ± 1.11	4.13 ± 0.82	0.176	0.007
Men	4.30 ± 0.95	4.11 ± 1.13	4.51 ± 0.68		
To what extent do you feel that physical pain prevents you from doing what you need to do? ^3^					
Women	1.33 ± 0.58	1.31 ± 0.65	1.35 ± 0.54	0.145	0.008
Men	1.34 ± 0.57	1.42 ± 0.62	1.25 ± 0.52		
How much do you need any medical treatment to function in your daily life? ^3^					
Women	1.15 ± 0.42	1.24 ± 0.55	1.10 ± 0.34	0.357	0.003
Men	1.22 ± 0.67	1.23 ± 0.68	1.21 ± 0.65		
How much do you enjoy life? ^3^					
Women	4.17 ± 0.71	4.31 ± 0.79	4.09 ± 0.67	0.354	0.003
Men	4.17 ± 0.71	4.20 ± 0.73	4.14 ± 0.69		
To what extent do you feel your life to be meaningful? ^3^					
Women	4.16 ± 0.65	4.19 ± 0.71	4.15 ± 0.62	0.487	0.002
Men	4.28 ± 0.77	4.31 ± 0.86	4,25 ± 0.67		
How well are you able to concentrate? ^3^					
Women	3.89 ± 0.75	4.02 ± 0.82	3.81 ± 0.72	0.217	0.006
Men	4.01 ± 0.72	4.00 ± 0.78	4.02 ± 0.65		
How safe do you feel in your daily life? ^3^					
Women	4.16 ± 0.65	4.19 ± 0.71	4.15 ± 0.62	0.904	< 0.001
Men	4.28 ± 0.77	4.31 ± 0.86	4.25 ± 0.67		
How healthy is your physical environment? ^3^					
Women	3.91 ± 0.78	3.93 ± 0.75	3.90 ± 0.81	0.190	0.006
Men	3.98 ± 0.80	4.11 ± 0.80	3.82 ± 0.81		
Do you have enough energy for everyday life? ^4^					
Women	4.18 ± 0.64	4.16 ± 0.70	4.19 ± 0.61	0.893	< 0.001
Men	4.34 ± 0.61	4.34 ± 0.62	4.35 ± 0.61		
Are you able to accept your bodily appearance? ^4^					
Women	4.00 ± 0.70	3.79 ± 0.73	4.02 ± 0.69	0.407	0.002
Men	4.24 ± 0.69	4.28 ± 0.66	4.19 ± 0.72		
Have you enough money to meet your needs? ^4^					
Women	3.98 ± 0.79	4.14 ± 0.63	3.89 ± 0.88	0.261	0.005
Men	3.93 ± 0.82	3.94 ± 0.86	3.91 ± 0.77		
How available to you is the information that you need in your day-to-day life? ^4^					
Women	4.65 ± 0.50	4.79 ± 0.43	4.57 ± 0.54	0.093	0.010
Men	4.71 ± 0.46	4.72 ± 0.46	4.70 ± 0.46		
To what extent do you have the opportunity for leisure activities? ^4^					
Women	4.33 ± 0.70	4.43 ± 0.73	4.28 ± 0.68	0.089	0.010
Men	4.28 ± 0.69	4.22 ± 0.75	4.35 ± 0.61		
How well are you able to get around? ^1^					
Women	4.84 ± 0.39	4.78 ± 0.46	4.87 ± 0.34	0.198	0.006
Men	4.88 ± 0.33	4.89 ± 0.32	4.86 ± 0.35		
How satisfied are you with your sleep? ^2^					
Women	3.82 ± 0.91	3.64 ± 0.85	3.92 ± 0.95	0.030	0.017
Men	3.96 ± 0.85	4.05 ± 0.85	3.86 ± 0.86		
How satisfied are you with your ability to perform your daily living activities? ^2^					
Women	4.26 ± 0.72	4.36 ± 0.62	4.20 ± 0.78	0.197	0.006
Men	4.34 ± 0.58	4.32 ± 0.62	4.37 ± 0.53		
How satisfied are you with your capacity for work? ^2^					
Women	4.23 ± 0.71	4.28 ± 0.67	4.23 ± 0.71	0.717	< 0.001
Men	4.46 ± 0.65	4.52 ± 0.68	4.39 ± 0.61		
How satisfied are you with yourself? ^2^					
Women	3.75 ± 0.60	4.00 ± 0.62	3.94 ± 0.82	0.821	< 0.001
Men	4.14 ± 0.72	4.18 ± 0.79	4.09 ± 0.64		
How satisfied are you with your personal relationships? ^2^					
Women	4.02 ± 0.82	4.09 ± 0.76	3.98 ± 0.86	0.366	0.003
Men	4.08 ± 0.80	4.22 ± 0.79	3.93 ± 0.83		
How satisfied are you with your sex life? ^2^					
Women	3.58 ± 0.96	3.52 ± 0.98	3.62 ± 0.95	0.170	0.007
Men	3.58 ± 1.09	3.69 ± 1.11	3.46 ± 1.08		
How satisfied are you with the support you get from your friends? ^2^					
Women	3.99 ± 0.73	4.00 ± 0.62	3.98 ± 0.79	0.067	0.012
Men	3.85 ± 0.76	4.02 ± 0.72	3.67 ± 0.81		
How satisfied are you with the conditions of your living place? ^2^					
Women	4.13 ± 0.90	4.28 ± 0.89	4.04 ± 0.91	0.386	0.003
Men	4.14 ± 0.91	4.34 ± 0.95	3.91 ± 0.88		
How satisfied are you with your access to health services? ^2^					
Women	4.19 ± 0.81	4.22 ± 0.75	4.18 ± 0.84	0.654	0.001
Men	4.25 ± 0.75	4.31 ± 0.84	4.18 ± 0.66		
How satisfied are you with your transport? ^2^					
Women	4.25 ± 0.79	4.31 ± 0.63	4.22 ± 0.87	0.830	< 0.001
Men	4.36 ± 0.78	4.38 ± 0.83	4.33 ± 0.72		
How often do you have negative feelings such as blue mood, despair, anxiety, depression? ^5^					
Women	2.23 ± 0.83	1.95 ± 0.76	2.23 ± 0.83	0.261	0.005
Men	1.63 ± 0.88	1.54 ± 0.94	1.74 ± 0.81		

*Note*. Results are presented as mean ± SD*.* p – *p*-value for ANOVA test. η^2^ – effect size

^1^1 = very poor, 2 = poor, 3 = neither poor nor good, 4 = good, 5 = very good

^2^1 = very dissatisfied, 2 = dissatisfied, 3 = neither satisfied nor dissatisfied, 4 = satisfied, 5 = very satisfied

^3^1 = not at all, 2 = a little, 3 = a moderate amount, 4 = very much, 5 = an extreme amount

^4^1 = not at all, 2 = a little, 3 = moderately, 4 = mostly, 5 = completely

^5^1 = never, 2 = seldom, 3 = quite often, 4 = very often, 5 = always

**Table 3:** Mean Likert-Scores of the WHQOLBREF-Items Displayed by Race Distance

		Control Group	NURMI-Runners			
Question	Total	10 KM	HM	M/UM	p	η^2^
How would you rate your Quality of Life? ^1^						
Women	4.33 ± 0.58	4.33 ± 0.58	4.26 ± 0.62	4.44 ± 0.51	0.111	0.016
Men	4.48 ± 0.65	4.45 ± 0.58	4.59 ± 0.56	4.37 ± 0.79		
How satisfied are you with your health? ^2^						
Women	4.09 ± 0.93	3.93 ± 1.03	4.18 ± 0.89	4.37 ± 0.69	0.111	0.016
Men	4.30 ± 0.95	4.39 ± 0.68	4.30 ± 1.03	4.21 ± 1.04		
To what extent do you feel that physical pain prevents you from doing what you need to do? ^3^						
Women	4.67 ± 0.58	4.67 ± 0.58	4.72 ± 0.53	4.56 ± 0.70	0.314	0.008
Men	4.66 ± 0.57	4.85 ± 0.36	4.67 ± 0.56	4.51 ± 0.69		
How much do you need any medical treatment to function in your daily life? ^3^						
Women	4.82 ± 0.43	4.83 ± 0.42	4.89 ± 0.45	4.81 ± 0.40	0.746	0.002
Men	4.78 ± 0.66	4.82 ± 0.47	4.85 ± 0.63	4.67 ± 0.81		
How much do you enjoy life? ^3^						
Women	4.17 ± 0.71	4.13 ± 0.72	4.18 ± 0.73	4.26 ± 0.66	0.924	0.001
Men	4.17 ± 0.71	4.12 ± 0.60	4.11 ± 0.90	4.28 ± 0.55		
To what extent do you feel your life to be meaningful? ^3^						
Women	4.11 ± 0.78	4.05 ± 0.84	4.12 ± 0.76	4.26 ± 0.66	0.950	< 0.001
Men	4.31 ± 0.81	4.24 ± 0.71	4.30 ± 1.03	4.37 ± 0.62		
How well are you able to concentrate? ^4^						
Women	3.89 ± 0.76	3.87 ± 0.74	3.89 ± 0.80	3.93 ± 0.73	0.903	0.001
Men	4.01 ± 0.71	3.97 ± 0.59	3.96 ± 0.70	4.09 ± 0.81		
How safe do you feel in your daily life? ^4^						
Women	4.16 ± 0.66	4.04 ± 0.71	4.23 ± 0.60	4.37 ± 0.57	0.267	0.010
Men	4.28 ± 0.76	4.30 ± 0.68	4.26 ± 0.91	4.28 ± 0.67		
How healthy is your physical environment? ^4^						
Women	3.91 ± 0.78	3.75 ± 0.84	4.07 ± 0.73	4.04 ± 0.65	0.220	0.011
Men	3.98 ± 0.80	4.00 ± 0.71	4.07 ± 0.88	3.86 ± 0.77		
Do you have enough energy for everyday life? ^4^						
Women	4.18 ± 0.64	4.07 ± 0.70	4.28 ± 0.56	4.26 ± 0.59	0.888	0.001
Men	4.34 ± 0.59	4.18 ± 0.64	4.37 ± 0.53	4.44 ± 0.59		
Are you able to accept your bodily appearance? ^4^						
Women	4.00 ± 0.70	3.95 ± 0.70	4.00 ± 0.71	4.15 ± 0.72	0.349	0.008
Men	4.24 ± 0.64	4.15 ± 0.80	4.35 ± 0.53	4.19 ± 0.63		
Have you enough money to meet your needs? ^4^						
Women	3.98 ± 0.79	3.91 ± 0.87	4.00 ± 0.73	4.15 ± 0.66	0.826	0.001
Men	3.93 ± 0.82	3.79 ± 0.82	3.98 ± 0.86	3.98 ± 0.77		
How available to you is the information that you need in your day-to-day life? ^4^						
Women	4.65 ± 0.50	4.64 ± 0.51	4.68 ± 0.47	4.63 ± 0.57	0.242	0.010
Men	4.71 ± 0.45	4.55 ± 0.51	4.78 ± 0.42	4.77 ± 0.43		
To what extent do you have the opportunity for leisure activities? ^4^						
Women	4.33 ± 0.70	4.21 ± 0.76	4.51 ± 0.57	4.30 ± 0.72	0.481	0.005
Men	4.28 ± 0.68	4.00 ± 0.66	4.41 ± 0.69	4.35 ± 0.65		
How well are you able to get around? ^1^						
Women	4.84 ± 0.37	4.76 ± 0.43	4.91 ± 0.29	4.93 ± 0.27	0.394	0.007
Men	4.88 ± 0.33	4.85 ± 0.36	4.87 ± 0.34	4.91 ± 0.29		
How satisfied are you with your sleep? ^2^						
Women	3.82 ± 0.91	3.83 ± 0.95	3.81 ± 0.85	3.81 ± 0.96	0.530	0.005
Men	3.96 ± 0.85	4.12 ± 0.76	4.00 ± 0.81	3.79 ± 0.97		
How satisfied are you with your ability to perform your daily living activities? ^2^						
Women	4.26 ± 0.72	4.23 ± 0.75	4.18 ± 0.74	4.52 ± 0.58	0.226	0.011
Men	4.34 ± 0.57	4.21 ± 0.49	4.39 ± 0.54	4.40 ± 0.66		
How satisfied are you with your capacity for work? ^2^						
Women	4.23 ± 0.71	4.23 ± 0.67	4.18 ± 0.69	4.33 ± 0.88	0.884	0.001
Men	4.46 ± 0.61	4.45 ± 0.56	4.43 ± 0.62	4.49 ± 0.63		
How satisfied are you with yourself? ^2^						
Women	3.96 ± 0.72	3.95 ± 0.73	3.91 ± 0.66	4.11 ± 0.80	0.095	0.017
Men	4.14 ± 0.61	4.09 ± 0.77	4.28 ± 0.46	4.02 ± 0.60		
How satisfied are you with your personal relationships? ^2^						
Women	4.02 ± 0.82	4.05 ± 0.80	3.96 ± 0.84	4.04 ± 0.85	0.699	0.003
Men	4.08 ± 0.80	4.00 ± 0.71	4.11 ± 0.85	4.12 ± 0.82		
How satisfied are you with your sex life? ^2^						
Women	3.58 ± 0.96	3.64 ± 0.94	3.51 ± 1.04	3.59 ± 0.84	0.698	0.003
Men	3.58 ± 1.09	3.48 ± 1.15	3.54 ± 1.13	3.70 ± 1.01		
How satisfied are you with the support you get from your friends? ^2^						
Women	3.99 ± 0.73	3.87 ± 0.68	4.11 ± 0.80	4.07 ± 0.68	0.423	0.006
Men	3.85 ± 0.75	3.76 ± 0.61	4.04 ± 0.82	3.72 ± 0.73		
How satisfied are you with the conditions of your living place? ^2^						
Women	4.13 ± 0.90	4.03 ± 0.94	4.21 ± 0.90	4.22 ± 0.75	0.861	0.001
Men	4.14 ± 0.91	3.91 ± 0.98	4.20 ± 0.96	4.26 ± 0.79		
How satisfied are you with your access to health services? ^2^						
Women	4.19 ± 0.81	4.00 ± 0.85	4.39 ± 0.75	4.33 ± 0.68	0.403	0.007
Men	4.25 ± 0.75	4.18 ± 0.73	4.33 ± 0.70	4.21 ± 0.83		
How satisfied are you with your transport? ^2^						
Women	4.25 ± 0.79	4.24 ± 0.69	4.25 ± 0.87	4.30 ± 0.87	0.073	0.019
Men	4.36 ± 0.77	4.03 ± 0.85	4.54 ± 0.66	4.42 ± 0.76		
How often do you have negative feelings such as blue mood, despair, anxiety, depression? ^5^						
Women	3.80 ± 0.80	3.80 ± 0.82	3.65 ± 0.79	4.11 ± 0.70	0.109	0.016
Men	4.37 ± 0.65	4.39 ± 0.61	4.37 ± 0.68	4.35 ± 0.65		

*Note*. Results are presented as mean ± SD*.* 10 km – 10 Kilometer Control Group. HM – Half Marathon. M – Marathon. UM – Ultramarathon. p – p-value for ANOVA test. η^2^ – effect size

^1^1 = very poor, 2 = poor, 3 = neither poor nor good, 4 = good, 5 = very good

^2^1 = very dissatisfied, 2 = dissatisfied, 3 = neither satisfied nor dissatisfied, 4 = satisfied, 5 = very satisfied

^3^1 = not at all, 2 = a little, 3 = a moderate amount, 4 = very much, 5 = an extreme amount

^4^1 = not at all, 2 = a little, 3 = moderately, 4 = mostly, 5 = completely

^5^1 = never, 2 = seldom, 3 = quite often, 4 = very often, 5 = always

### Main effects of race distance on quality of life and diet×race distance interaction

Mean scores in physical health were 17.5 ± 1.5 (84.3%) in female members of the 10-km control group, 18.0 ± 1.1 (87.4%) in male members of the 10-km control group, 17.7 ± 1.3 (85.6%) in female half marathoners, 18.1 ± 1.2 (87.8%) in male half marathoners, 17.8 ± 1.3 (86.5%) in female marathoners/ultramarathoners and 17.8 ± 1.6 (86.4%) in male marathoners/ultramarathoners. In terms of psychological wellbeing, mean scores were 15.8 ± 2.0 (73.9%) in female members of the 10-km control group, 16.7 ± 2.0 (79.1%) in male members of the 10-km control group, 15.8 ± 2.1 (74.0%) in female half marathoners, 16.9 ± 2.0 (80.7%) in male half marathoners, 16.5 ± 2.1 (78.4%) in female marathoners/ultramarathoners and 16.9 ± 1.6 (80.4%) in male marathoners/ultramarathoners. Mean scores in social relationships were 15.4 ± 2.6 (71.3%) in female members of the 10-km control group, 15.0 ± 2.6 (68.7%) in male members of the 10-km control group, 15.4 ± 2.8 (71.5%) in female half marathoners, 15.6 ± 3.0 (72.4%) in male half marathoners, 15.6 ± 2.5 (72.5%) in female marathoners/ultramarathoners and 15.4 ± 1.7 (71.1%) in male marathoners/ultramarathoners. With regard to environment, mean scores were 16.4 ± 1.6 (77.6%) in female members of the 10-km control group, 16.4 ± 1.8 (77.4%) in male members of the 10-km control group, 16.4 ± 1.5 (77.6%) in female half marathoners, 17.28 ± 1.7 (83.0%) in male half marathoners, 17.2 ± 1.3 (82.3%) in female marathoners/ultramarathoners and 17.1 ± 1.7 (81.6%) in male marathoners/ultramarathoners.

No major effect of race distance on physical health (*p* = 0.586, η^2^ = 0.007 and *p* = 0.847, η^2^ = 0.003), psychological wellbeing (*p* = 0.379, η^2^ = 0.013 and *p* = 0.818, η^2^ = 0.003), or social relationships (*p* = 0.986, η^2^ < 0.001 and *p* = 0.838, η^2^ = 0.003) for women and men, respectively was shown.

Also, no effect of race distance on environment for men was found (*p* = 0.121, η^2^ = 0.036). However, a minor effect was observed for women (*p* = 0.014, η^2^ = 0.054), where half-marathoners had a higher environment score than the members of the 10-km control group **(**Fig. 2a, 2b, Table 3).

No diet×race distance interaction on physical health (*p* = 0.346, η2 = 0.014 and *p* = 0.060, η2 = 0.047), psychological well-being (*p* = 0.672, η2 = 0.005 and *p* = 0.026, η2 = 0.061) or social relationships (*p* = 0.490, η2 = 0.009 and *p* = 0.112, η2 = 0.037) for women or men, respectively, was observed. A moderate diet×race distance interaction on environment score was shown for men (*p* = 0.013, η2 = 0.072), but no interaction was found for women (*p* = 0.925, η2 = 0.001).

## Discussion

This study aimed to investigate QOL of female and male endurance runners following a vegetarian or vegan diet and to compare it to female and male endurance runners adhering to an omnivorous diet. The hypothesis was that QOL would be equal in both groups and hence a vegetarian or vegan diet could be an equivalent alternative to an omnivorous diet.

The main findings were that *(i)* men had higher scores in physical health and psychological well-being as compared to women, but there were no sex differences in terms of social relationships counts and environment scores, *(ii)* no major effect of diet on physical health and psychological wellbeing in either sex, on social relationships for women or on environment for men, was observed, *(iii)* a minor effect of diet on social relationships for men and environment for women was shown, with higher score for omnivores, *(iv)* no major effect of race distance on physical health, psychological and social relationships was shown for either women or men, *(v)* no effect of race distance on environment for men was found, but a minor effect was observed for women, where half-marathoners had a higher environment score than the members of the 10-km control group, *(vi)* no diet×race distance interaction on physical health, psychological wellbeing or social relationships was observed for women or men, and *(vii)* a moderate diet×race distance interaction on environment score was shown for men, although no interaction was found in women.

### Sex differences in quality of life

A first important finding was that male endurance runners have higher overall QOL scores compared to female endurance runners, mainly based on higher counts in the domains of physical health and psychological wellbeing. These sex differences have been observed in other studies as well [11, 16, 17], particularly relating to psychological factors [37].

A potential explanation could be that women are more emotional and sensitive to perceived pressure, as compared to men [38, 39]. It has been shown that women are more willing to report symptoms [40] whereas men often stick to traditional role concepts. They think society expects them to be strong and self-reliant (‘Macho-Concept’, ‘Social desirability’), but they must not complain about symptoms or other ‘sissy-stuff’ [41, 42]. The phenomenon that women report poorer (physical) health is well known and is termed ‘gender paradox’. Although women live longer than men on average, researchers have found that women are more likely to report poorer health, suffer higher rates of morbidity, and use more health services than men [43, 44]. In terms of social relationship scores, there were no detectable differences between men and women, which contradicts results of previous studies [17, 18]. This can be explained by the fact that athletes usually have higher scores in this domain and thus any sex difference was eliminated [45]. In environment scores, there were no sex differences either. This finding is consistent with the results from other research [14].

### Impact of the choice of the diet on quality of life

A second important finding was that diet choice does not affect the QOL-domains of physical health, psychological wellbeing, and social relationships for women or environment for men. However, our subjects showed that mean total domain scores are constantly high level (i.e. 16.99 on the 4–20 scale), mainly exceeding scores that have been generated for the general population in other studies (i.e. 15.70 [46] and 15.22 [47] on the 4–20 scale).

These findings confirmed our hypothesis that QOL of runners who adhere to a vegetarian or vegan diet is as good as the QOL of those who follow an omnivorous diet. Thus, they supported the notion that a vegetarian or vegan diet can be an appropriate and an equivalent alternative to an omnivorous diet.

The results are consistent with current research. Several studies have shown high QOL scores in vegetarians [13] and vegans [10, 48]. A reasonable explanation is the fact that a diet rich in fruit and vegetables leads to a higher degree of fitness and lower morbidity, and thus to a good health status [5, 7, 49]. It is beyond debate that a healthy body is an inevitable requirement for a healthy mind – and hence for a high perception of QOL [50]. The dictum ‘Mens sana in corpore sano’ – ‘a healthy mind in a healthy body’ – takes up this idea and also applies vice versa. This assumption has been supported by studies showing that vegetarians and vegans report low stress levels and good states of mood [21, 22].

Moreover, the high QOL scores can be explained by the personality profiles as well as moral concepts and personal beliefs of vegetarians and vegans. A current investigation shows that they tend to be more liberal, altruistic, universalistic, and empathic [48] and often deal intensively with moral and ethical concerns relating to animal treatment and conscious behavior towards the environment [49]. This could make them believe that they contribute to a sustainable relationship between mankind and environment [50], which could generate a higher life satisfaction.

However, we found a minor effect of diet on social relationships scores for men. This result can be explained again by men’s self-perception or awareness of other men. The fact that men often still stick to traditional role concepts [41, 42] could lead them to consider male vegetarians or vegans as not being real men, since a real man has to eat meat [51]. This would evoke the impression of being isolated and excluded, consequently leading to a reduction in self-esteem and thus to lower life satisfaction. In addition, current literature reveals that vegetarians and vegans more often report that they neither live with a partner nor are married, respectively [52, 53]. This tendency could be identified in our sample as well. Since it is well known that having a girlfriend/boyfriend or wife/husband leads to a certain degree of life satisfaction [54] and, beyond that, prevents affective disorders such as depression [55], this fact could have caused lower scores as well.

Furthermore, our female subjects who adhered to an omnivorous diet had higher environment scores than the vegetarians/vegans. This finding was surprising because it was not consistent with existing literature. Since consumption of fruits and vegetables and thus vegetarianism/ veganism is regarded to be associated with a good socioeconomic background [56], we had expected that this would lead to high scores in financial resources, access to health and social care, and opportunities for acquiring new information and skills, which are the facets incorporated in the dimension environment. However, our subjects may have considered other facets in this dimension, for example, freedom, physical safety and physical environment, to be more important. As vegetarians and vegans usually have high demands concerning these topics, especially in the matter of physical environment [23, 57], this might have made them state lower satisfaction in this regard.

### Impact of the race distance on quality of life

A third important finding was that our data did not show an interaction between race distance and physical health, psychological well-being and social relationships for women men.

In addition to the fact that mean QOL-scores of our subjects were consistently high, these results suggest that endurance running leads to a high degree of life satisfaction, regardless of the race distance. The findings are consistent with other research results [33, 58, 59]. There are various reasons which could explain this.

Similar to a well-balanced diet, physical activity in general, and endurance running in particular, are crucial factors which affect health. In this context, the ‘healthy mind in a healthy body’-concept, which has already been mentioned before, could again provide an explanation [60, 61]. Research into endocrine responses to exercise has shown a positive correlation between endurance training and endorphin levels [62]. Since endorphins are regarded to be responsible for good mood and a reduction in sensation of pain [63], these changes lead to a lower level of perceived stress and thus to well-being. Similar tendencies can be found for stress and anxiety perception in athletes. Endurance running in particular leads to a higher resilience to stress and anxiety [64], a good sleep architecture [30], and an increased self-perception specifically in terms of a perceived internal and body competence [65]. As both the NURMI-Runners and the members of the 10-km control group derived high scores in the physical and psychological well-being dimensions, it appears likely that the previous explanation applies to both groups.

Besides health, sleep and body consciousness, motivational concerns and personality profiles of endurance runners are the basis for their high life satisfaction. Most athletes run voluntarily and therefore they are motivated by intrinsic reasons, such as self-esteem, self-discovery, improved fitness, life meaning or personal goal achievement and challenge [66]. Since endurance running challenges both body and mind to an extreme degree [67, 68], finishing a marathon shows that someone can achieve her/his goals and knows or even expands her/his personal limitations or abilities. In this context, the ability of ‘self-conquest’ is a crucial factor that contributes to the perception of extraordinary and wonderful feelings, leading to a certain degree of happiness and hence high QOL scores [12]. Furthermore, several authors have investigated the personality profiles of endurance athletes. They were described as task-oriented rather than ego-oriented, health and financially conscious [69], extroverted [70] and self-sufficient [71]. Moreover, they would have a certain degree of emotional intelligence [72]. These character traits are typically regarded to be positive and thus have positive effects on social relationships – one dimension of the QOL-domains. Since there were no detectable differences between the NURMI-Runners and the members of the 10-km control group in this regard, our findings suggest that these character traits apply to endurance runners of any distance and are not limited to one subgroup.

Furthermore, our data demonstrated a minor effect of the race distance on environment scores for women, where half-marathoners had higher counts than the members of the 10-km control group. Considering that the domain of environment was assessed using, among others, the categories financial resources, freedom and security, home environment, participation in leisure activities, and transport, the finding could be explained by the socioeconomic background of the related athletes. It has been reported that marathon runners tend to have an above average high socioeconomic status [2, 73]. Belonging to a high social class means having more financial resources, a better home environment and better access to transport.

Summarizing the effects of diet choice and race distance on QOL, it can be concluded that the dual approach of regular physical activity, i.e. endurance running, and conscious nutrition, i.e. a vegetarian/vegan diet, is a crucial factor in the derivation of the high QOL scores that were found in the subjects. Beyond that, these two factors are synergistic and thus mutually reinforcing [23], which increases their impact. Obviously, the positive effects of endurance running doesn’t seem to depend on the race distance, as both of the NURMI-Runners and the 10-km controls showed high QOL scores. Further research is warranted to determine the optimal balance within the dual approach of physical activity such as endurance running linked to vegetarian or vegan nutrition, in order to achieve cumulative effects [23] for a high QOL.

### Diet×race-distance-interaction and its impact on quality of life

A fourth important finding was that our data did not reveal a diet***×***race distance interaction concerning physical health, psychological wellbeing or social relationships for women or men.

Diet choice immediately before running or the composition of the personal diet might be influenced by the announced race distance [74, 75]. However, there is no evidence that the choice of diet in general has an effect on the preferred race distance and vice versa. Thus, an interference of one of the variables with the other affecting the influence on QoL would have been unexpected.

Nevertheless, a moderate diet***×***race distance interaction on the environment score was shown for men, although no interaction was found for women. This result could again be explained by the socioeconomic background of the runners. As has already been mentioned above, marathon runners tend to have above average levels of intelligence quotient (IQ) and a high socioeconomic status [2, 73]. High IQ scores [76, 77] and belonging to a high socioeconomic group is positively correlated with the ability to reflect critically about diet choice [78, 79]. In this way, an interaction between diet choice and race distance is possible.

### Limitations and implications for future research

Some limitations of our study should be noted. The survey is based on self-report, meaning that the reliability of the data depends on the conscientiousness of our subjects. However, we minimized this effect by using questions to control for diet and race distance.

Moreover, the small sample size and the pre-selection of our subjects, due to the fact that only highly motivated runners took part, led to a lack of statistical representativeness, which might have affected our results. Nonetheless, the high intrinsic motivation of the participants would have led to an increase in the accuracy of their answers and hence to a higher quality of the generated data.

### Practical applications

Since our survey is the first to investigate QOL in endurance runners adhering to a vegetarian or vegan diet, the results might be important for researchers involved in implementing individualized dietary strategies for athletes and thus may be used as reference for future studies. Moreover, our data may support recreational and professional runners as well as their coaches in finding an optimized nutrition strategy. Not only athletes but also non-runners and physicians might get a better insight into appropriate diets and more active lifestyles, and thus have a better basis for their choices for themselves, their families and even their patients. Beyond that, in the light of the aforementioned dual approach of regular physical activity integrated with vegetarian/vegan nutrition providing cumulative benefits for a high level of life satisfaction, the results might be used as a basis for public health and prevention programs for both children and adults.

## Conclusion

In summary, our results reveal that the participants of our study, including the members of the 10-km control group as well as the NURMI-Runners, had a high QOL, regardless of the race distance or diet choice. These findings contribute to a broad body of evidence supporting the notion that adhering to a vegetarian or vegan diet can be an appropriate and equal alternative to an omnivorous diet. In combination with an active lifestyle, i.e. by performing regular endurance running, this dual approach can be one way to effectively and successfully achieve a high degree of life satisfaction.

## Abbreviations

10 km: 10-Kilometer Control Group
DOM: Domain
EKSG: Ethics Board of St. Gallen, Switzerland
HM: Half Marathon
M: Marathon
NURMI: Nutrition and Running High Mileage
QOL: Quality of Life
SD: Standard Deviation
UM: Ultramarathon
WHOQOLBREF: World Health Organization Quality of Life Assessment - brief version (french: bref)
